# Development of a GPCR-binding ligand screening method using real-time interaction analysis on live cells

**DOI:** 10.1016/j.bbrep.2026.102818

**Published:** 2026-09-30

**Authors:** Hadis Westin, Hanna Yushchyshyna, Olga Vorontsova, Saloni Chopra, Tianqi Xu, Christoffer Bengtsson, Fabio Begnini, Preeti Jha, Ulrika Rosenström, Ulrika Yngve, Marika Nestor, Jos Buijs

**Affiliations:** a Ridgeview Instruments AB, Uppsala, Sweden; b Department of Immunology, Genetics and Pathology, SciLifeLab, Uppsala University, Uppsala, Sweden; c Drug Discovery and Development Platform, SciLifeLab, Sweden; d Department of Biochemistry & Biophysics, Stockholm University, Solna, Sweden; e Department of Medicinal Chemistry, Uppsala University, Uppsala, Sweden

**Keywords:** GPCR ligand interactions, Theranostic ligand, Neurokinin 1 receptor (NK1-R), Live-cell screening assay, Targeted radiotherapy, Receptor binding kinetics, Binding affinity

## Abstract

G protein-coupled receptors (GPCRs) affect many physiological processes and frequently used as drug targets and biomarkers. The present study focuses on the neurokinin 1 receptor (NK1-R) which is over-expressed on neural tumours with the aim to develop high affinity binders to NK1-R for theranostic purposes. 60 Ligands were synthesized, all containing iodine enabling isotopic exchange with radioactive iodine for imaging or radiotherapeutic applications. Embedded in the cellular membrane, GPCRs adopt various conformational states that can change upon interaction with ligands. Therefore, a time-resolved, live cell assay was developed to evaluate competition with fluorescently labelled endogenous NK1-R ligand, Substance P (FAM-SP). The time resolved assay enabled quantification of the efficiency in which the ligands compete for the rapid and slow releasing NK1-R binding modes of FAM-SP. Of the 60 ligands, 25 were able to displace FAM-SP faster than the anti-nausea drug aprepitant. Two of these ligands displaced the rapid releasing FAM-SP fraction faster than Substance P (SP) and one of them also replaced the slow releasing fraction faster than aprepitant. To validate the assay, the affinity of two ligands for several NK1-R expressing cell lines was further quantified; one that had similar competition characteristics as SP and one that behaved as aprepitant. The ligand with similar competition characteristics as SP displayed a low nM to sub-pM affinity with a stronger binding correlating with higher receptor expression levels. This high affinity makes it a highly promising candidate for further development into a theranostic agent to address neural tumours.

## Highlights

• A live cell screening assay was developed to study competition of two NK1R – Substance P binding modes.
• 27 out of 60 iodine containing ligands competed similarly to or better than aprepitant.
• The lead compound has high affinity for NK1R expressing cells – a promising candidate for imaging or radiotherapy.

## Graphical abstract

**Figure:**
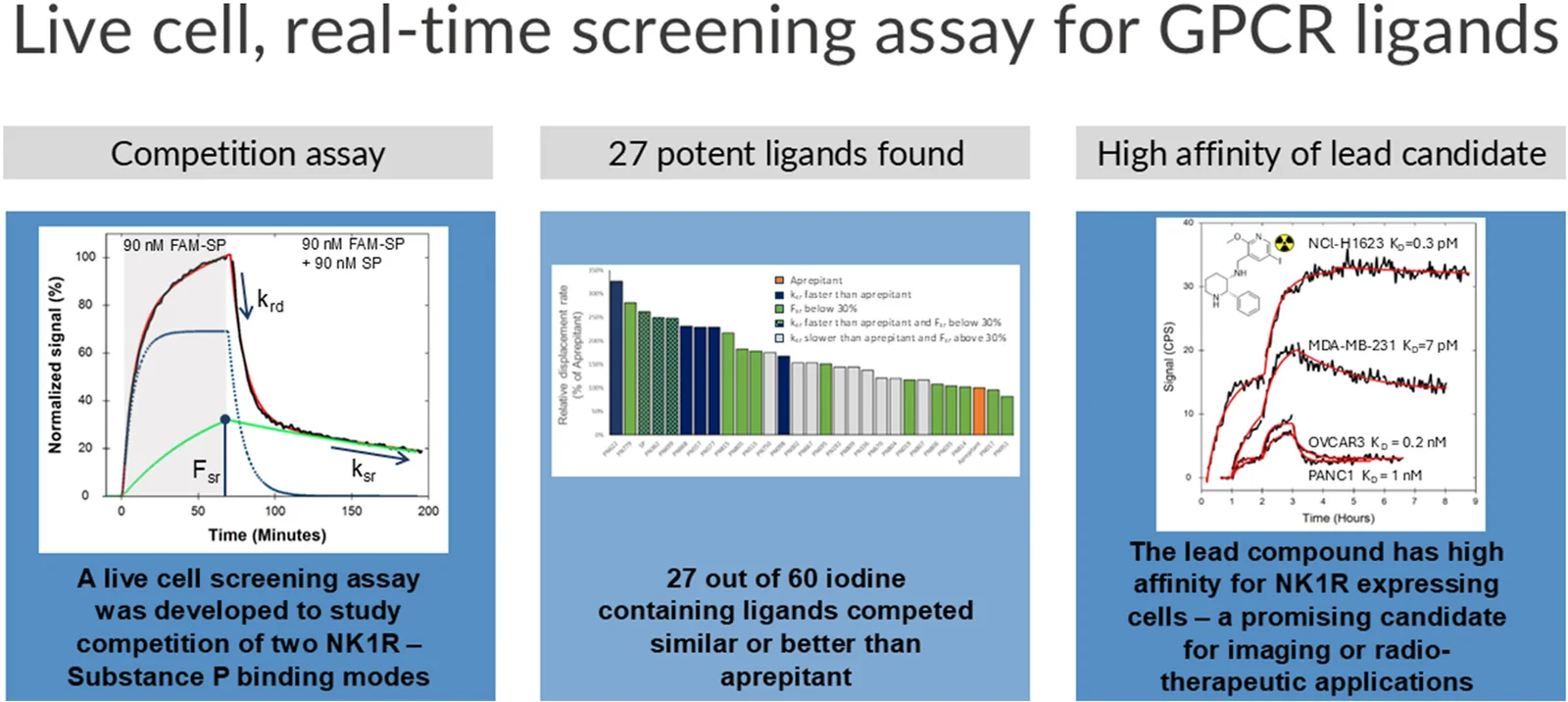

## 1 Introduction

G protein-coupled receptors (GPCRs) are the most prominent drug targets due to their central role in numerous physiological processes [1]. Accordingly, more than one-third of drugs that are FDA-approved or currently in clinical trials target GPCRs [2].

Neurokinin 1 receptor (NK1-R), also known as the Substance P receptor, is a G protein-coupled receptor located in the central nervous system (CNS) and peripheral tissues. NK1-R is a member of the tachykinin receptor family and is primarily activated by Substance P (SP) that plays a crucial role in processes like pain transmission, inflammation, vomiting, and stress responses [3]. NK1-R antagonists, such as aprepitant (oral administration) and fosaprepitant (intravenous administration), are used for treatment of chemotherapy-induced nausea and vomiting (CINV) [4]. SP can also act as a mitogen, promoting tumorigenesis and metastasis by stimulating cytokine release in the tumour microenvironment, facilitating tumour progression [5,6].

SP interacts with several transmembrane domains at the extracellular face of the receptor [7] with high, sub-nanomolar, affinity [8]. The SP binding induced formation of the binding pocket leads to G-protein coupling and activation of the receptor [8]. After prolonged activation, NK1-R undergoes desensitization through receptor internalization leading to a state where a substantial fraction of receptors resides intracellularly [8,9]. Internalized receptors may either be recycled back to the membrane or degraded in lysosomes [[10], [11], [12]].

Overexpression of NK1-R on neural tumours such as glioblastoma and neuroblastoma as well as in other cancers such as lung cancer, breast cancer, and osteosarcoma has made it a highly interesting target for theranostic applications. Since the NK1-R overexpression on tumour cells plays a crucial role in carcinogenesis by modulating apoptosis, angiogenesis, and migration, targeted radionuclide imaging and therapy is a promising approach for personalized diagnosis and therapy of cancer patients with NK1-R positive tumours [[13], [14], [15]].

In earlier studies, chelator-conjugated SP have been developed for theranostic applications [[16], [17], [18]]. However, rapid *in vivo* degradation remains a challenge, potentially affecting targeting efficiency and biodistribution [19,20]. SP also binds to other tachykinin receptors, such as NK2-R and NK3-R, which can lead to off-target effects and unintended physiological responses. Consequently, modified SP analogues and metabolically more stable nonpeptidic NK1-R antagonists are favoured for theranostic purposes [15,[21], [22], [23]]. Such alternative ligands may provide enhanced stability and specificity, thereby reducing the widespread physiological effects linked to NK1-R activity and improving their suitability for therapeutic use. The NK1-R antagonist aprepitant has been extensively studied for theranostic applications [[24], [25], [26], [27]] including radiolabelled aprepitant derivatives [26]. However, aprepitant's high lipophilicity results in suboptimal biodistribution which limits its potential. Therefore, several studies have focused on synthesizing novel NK1-R binders for imaging purposes [19,28]. Still, increased specificity and hydrophilicity to further limit uptake by lungs and liver would be beneficial. To this end, 60 ligands based on the phenylpiperidine scaffold were synthesized with the aim of improving theranostic applications. These new ligands all contain iodine that can be replaced with a radioactive iodine isotope suitable for imaging and radiotherapy applications.

To access the suitability of ligands for receptor binding in drug discovery, a combination of binding assays, biophysical methods, and cell-based assays are used [29,30]. As GPCRs exist is inactive and active conformations that are related to intracellular G-protein coupling [[7], [8], [9], [10],[31], [32], [33]], cell based binding assays are more suitable than biophysical binding assays. Moreover, the shift towards the active conformation upon ligand binding [[33], [34], [35]] and internalization and recycling processes [9,12,35,36] require that the binding assay considers the changing binding mode as function of ligand incubation time.

The need for kinetic screening in combination with a cellular environment for GPCRs has been recognized and has for example led to the development of a dual timepoint competition association assay to screen for 35 ligands against the adenosine A1 receptor using cell homogenates [37]. To design a competition assay with higher time-resolution and a live-cell environment, LigandTracer technology was applied. This technology is typically used for real-time binding studies on living cells to provide detailed insights into binding kinetics and affinities and, for example, used to unravel complex binding patterns of ligand – GPCR interactions [35,38]. As the throughput of LigandTracer is limited to a few assays per day, ligand screening based on time resolved analysis of the affinity and kinetics of the interaction has primarily been performed with smaller libraries to select and characterize novel ligands, such as antibodies [39,40], proteins [41,42], and small molecules [43,44]. A similar time resolution and throughput can be obtained with alternative technologies such as SPR imaging [45], quartz crystal microbalance [46,47], and single cell interaction cytometry [48,49]. However, to reduce signal fluctuation and cell degradation with these alternative technologies, experiments are often performed with fixated cells and limited assay duration [45].

For ligands that bind receptors in a simple one-to-one mode, where each ligand engages with the receptor in a similar manner, competition assays can be evaluated using commercially available models. Since SP interacts with NK1-R through a more complex binding mechanism, we developed a novel evaluation method to characterize competition efficiency of ligands for the various SP – NK1-R states within a single assay. This approach enabled monitoring competition of the 60 new ligands with SP on live cells, with NK1-R in its native membrane environment, at high temporal resolution. The competition efficiency of the 60 compounds was compared to that of aprepitant. The performance of the screening assay was validated by investigating the interaction of two selected representative ligands with several NK1-R expressing cancer cell lines.

## 2 Material and methods

### 2.1 Cell culture

HiTSeeker cell lines expressing human Tachykinin receptor 1 (TACR1, DNA Accession Number: GenBank: AY462098) in U2OS cells, TACR1 (NK1)/U2OS (U2OS-NK1R), and HEK 293 cells, TACR1 (NK1)/HEK293 (HEK293-NK1R) were obtained in cryopreserved vials from InnoProt, Spain (Cat# P30129-U2OS and P30129-HEK293). All cell media was supplemented with fetal bovine serum (FBS, Merck, Cat# F7524) and 100 I.U./ml penicillin–100 μg/mL streptomycin solution (PEST) obtained from ThermoFisher/Gibco (Cat# 15140-122). U2OS-NK1R and HEK293-NK1R were cultured in Dulbecco's modified Eagle medium (DMEM, 4.5 g/L glucose, GlutaMAX™ Supplement, pyruvate, Cat# 31966021), supplemented with 10% FBS and 1% PEST. To maintain NK1-R expression, 500 μg/mL G-418 (ThermoFisher/Gibco, Cat# 10131-027) was added to the cell culture medium for U2OS-NK1R cells and 50 μg/mL puromycin (ThermoFisher/Gibco, Cat# A113803) for HEK293-NK1R cells.

To characterize the interaction affinity and kinetics of 2 ligands, various NK1-R expressing cancer cell lines were used: MDA-MB-231 (ATCC, Cat# HTB-26), OVCAR3 (ATCC, Cat# HTB-161), PANC1 (ATCC, Cat# CRL-1469), and NCl-H1623 (ATCC, Cat# CRL-5881). MDA-MB-231, OVCAR3, and NCl-H1623 was cultured in RPMI1640 with stable glutamine (Biowest, Cat# L0498-500) supplemented with1% PEST and 20%, 10%, and 5% FBS, respectively. OVCAR3 medium also contained 0.01 mg/mL insulin (ThermoFisher/Gibco, Cat# 12585014). PANC1 was cultured in DMEM supplemented with 10% FBS and 1% PEST.

Cells were grown in tissue culture flasks (VWR, Cat. #734-2313) in a humidified incubator at 37 °C with 5% CO_2_. At 80%–90% confluency, cells were trypsinised using Trypsin-EDTA solution (ThermoFisher/Gibco, Cat# 25200-056), re-suspended, counted, 10-fold diluted, and transferred into a new flask.

For real-time cell binding and competition assays, 1-2 × 10^6^ cells in 3 mL cell culture medium were seeded into Petri dishes (Nunc™ Cell Culture/Petri Dishes, ThermoFisher Cat# 150350) that were placed tilted on the LigandTracer cell dish holder to only partially cover the dish area and incubated at 37 °C and 5% CO_2_ overnight. The next day the medium was replaced with 10 mL fresh medium, and the dishes were incubated overnight horizontally. Before every binding and competition assay with overexpressing U2OS-NK1R and HEK293-NK1R cells, medium was replaced with 3 mL CO_2_-Independent medium (Gibco, Cat# 18045-054) supplemented with 10% FBS. All cell binding assays with endogenous NK1-R expressing cell lines were performed with 3 mL of the same medium used for cell culturing.

### 2.2 NK1-R-binding ligands

In an iterative process, 60 *de novo* small molecules aiming to bind to the NK1-R were designed and synthesized. All ligands are based on the phenylpiperidine scaffold and contain a stable iodine that can be replaced with a radioactive iodine isotope suitable for imaging and therapy. For the design of all ligands, solubility, plasma protein binding and metabolic stability were considered. The ligands can be labelled using either direct iodination (PN951) or ipso-demetallation iodination reactions. The general structure of the ligands is shown in Fig. 1 and a few specific examples in S1. The exact chemical structures and synthesis of all ligands will be described elsewhere. The initial purity of the 60 ligands was >95% for 56 ligands, 4 ligands had a purity of 90-92% as inferred from HPLC and NMR analysis.

**Fig. 1:**
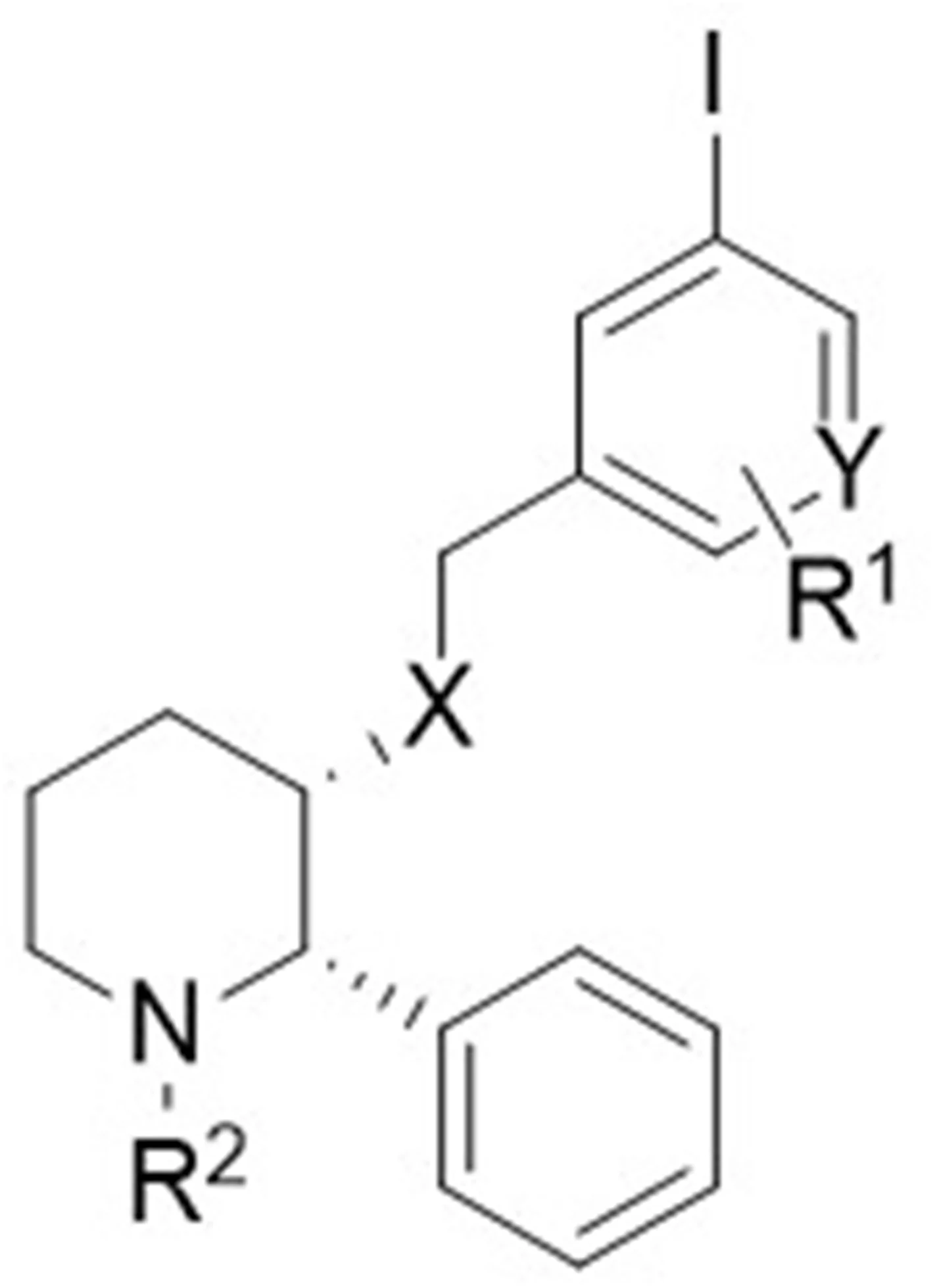
General structure of the ligands. X is N or O; Y is C or N, R^1^ is for example CF_3_ or OMe, R^2^ is H or for example -CH_2_-triazolone.

### 2.3 ^125^I-labeling of lead compound

To validate the screening assay, two ligands were labelled with ^125^I to quantify the affinity and kinetics of their interaction with NK1-R expressing cells. The chemical structures of these ligands, together with that of aprepitant, are given in supplementary information (Fig. S1). In general, ligands are radio-iodinated using oxidative iododestannylation with Chloramine-T as the oxidant according to a procedure published previously [50], see Fig. 2. The final ligand was isolated using preparative HPLC, reformulated into a suitable solvent, and analysed by HPLC using UV and radio detectors. The radiochemical purity was above 97%. The concentration of the ligands was calculated based on the specific activity of [^125^I]NaI.

**Fig. 2:**
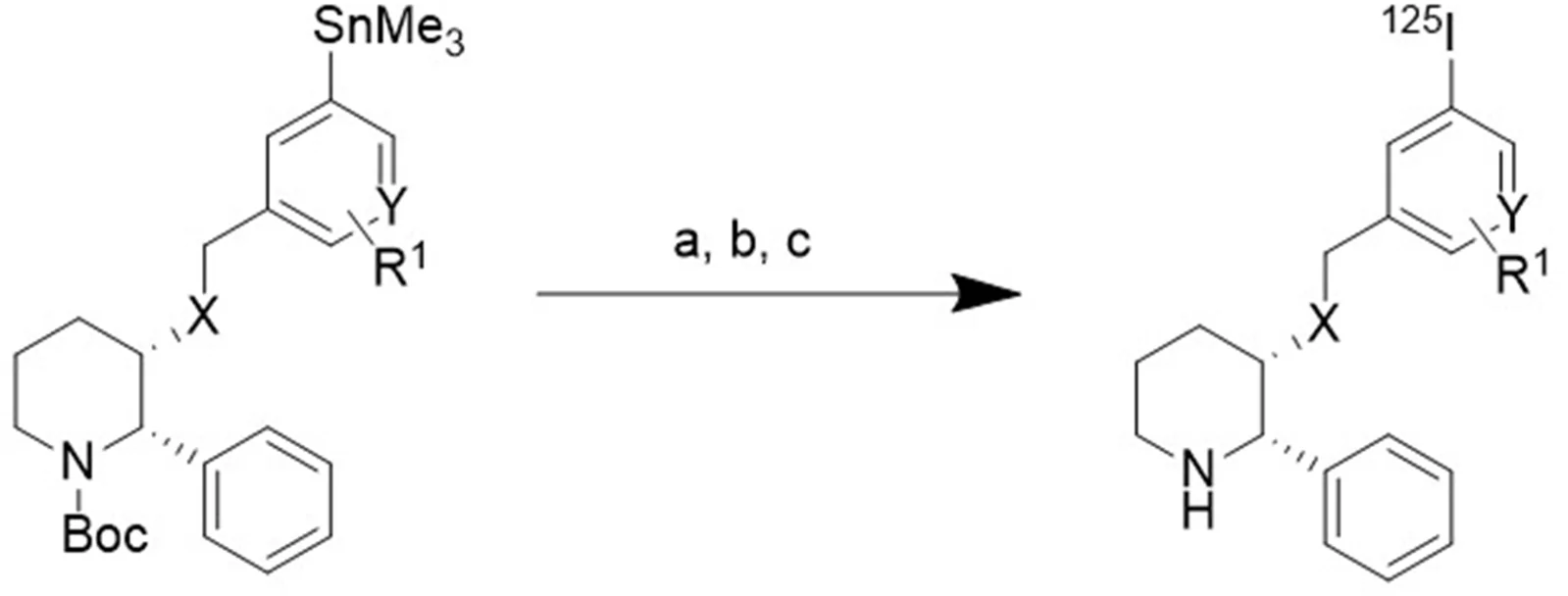
a) Chloramine-T, [^125^I]NaI, 5% HOAc in MeOH, 50 °C for 30 min, b) Na_2_S_2_O_5_ c) TFA, 65 °C 20-60 min. Boc (tert-Butyloxycarbonyl) is used as protective group during radio-iodination when the R^2^ group is H.

### 2.4 Binding kinetics and competition studies

The interaction of FAM-labelled SP (FAM-SP, AnaSpec, USA) with NK1-R on live cells was monitored in real-time cell binding assay (RT-CBA) or time resolved screening assay, using LigandTracer Green (Ridgeview Instruments AB, Cat# 1-04-002) equipped with a BlueGreen detector (Cat# 1-04-101). RT-CBA assays with ^125^I-labelled ligands were performed using LigandTracer Grey (Ridgeview Instruments AB, Cat# 1-01-001).

In brief, a LigandTracer assay is performed at ambient temperature by placing a Petri dish with cells seeded in the target area and 3 mL medium, on the inclined, rotating support of the LigandTracer instrument. Default settings for the one target assay (detection in target and background position for 30s each and a 5 s detection delay time) were used for all assays with cells in the target position and plastic of the dish, coated with adsorbed serum proteins from FBS, in the background position. During each dish rotation, bulk liquid flows to the lower part of the dish allowing detection of signals coming either from cell-associated ligands in the target area or nonspecific binding (if any) to the background area. For all assays, baseline signals for both positions are measured for 15-30 min before adding ligands. This baseline is used to align binding curves to start at zero signal before ligand is added. Unless stated otherwise, presented binding curves are based on the signals in the target area minus the signal in the background area. For RT-CBA, the baseline measurement is followed by incubating two or three consecutive ligand concentrations, for approximately 1 h per concentration, that are in the same range as the affinity of the interaction. Thereafter, the ligand containing medium was replaced with fresh medium to monitor dissociation. For competition assays, Petri dishes containing U2OS-NK1R cells were first incubated with 90 nM FAM-SP for 1 h followed by the addition of an equimolar amount of unlabelled SP, aprepitant, or an in-house synthesized ligand, where the displacement/replacement of FAM-SP was monitored for at least an hour.

### 2.5 Data analysis

Kinetic interaction evaluations were performed using TraceDrawer 1.9.2 and InteractionMap analysis (Ridgeview Instruments AB, Sweden) as described previously for ligand binding to GPCRs [51]. To analyse FAM-SP interactions with U2OS-NK1R and HEK293-NK1R cells both the OneToTwo (1:2) and OneToOneTwoState (1:1*) models were applied.

The simplest interaction model that describes the reversible interaction between a ligand (L) in solution and a target (T) expressed on the cell surface, is the so-called 1:1 Langmuir binding model (Eq. (1)) with an association rate constant (k_a_) for complex formation and a single dissociation rate constant (k_d_) for complex decay. The ratio of the rate constants k_d_/k_a_ gives the affinity K_D_.

(Eq. 1) $$\left[L\right]+{\left[T\right]\;}_{\underset{k_{d}}{\leftarrow }}^{\overset{k_{a}}{\to }}\;\left[LT\right]$$

More complex interaction models are required to describe heterogenous interactions with more than one k_a_ and k_d_. For example, the 1:2 model assumes that the ligand can bind to two different types of target populations, resulting in two independent 1:1 interactions with distinct kinetic parameters. The relative contribution of these two interactions is calculated from the signals that correspond to target saturation (B_max_), a value that is calculated when applying the model.

(Eq. 2) $$\left[L\right]+{\left[T_{1}\right]\;}_{\underset{k_{d1}}{\leftarrow }}^{\overset{k_{a1}}{\to }}\;\left[LT_{1}\right]\;and\;\left[L\right]+{\left[T_{2}\right]\;}_{\underset{k_{d2}}{\leftarrow }}^{\overset{k_{a2}}{\to }}\;\left[LT_{2}\right]$$

The 1:1* model assumes that after binding to the target, the bound complex can go to a second, stabilized state, in a reversible manner.

(Eq. 3) $$\left[L\right]+{\left[T\right]}_{\;\underset{k_{d1}}{\leftarrow }}^{\;\overset{k_{a1}}{\to }}\;{\left[LT\right]\;}_{\underset{k_{d2}}{\leftarrow }}^{\overset{k_{a2}}{\to }}\;\left[{LT}^{*}\right]$$

For this model, the affinity of the second process, K_D2_ is calculated from the ratio between the rate constants (k_d2_/k_a2_) and reflects the ratio between non-stabilized [LT] and stabilized [LT*] concentrations in equilibrium.

For evaluation of the competition assays, the curves were first normalized by rescaling the signal of the 90 nM FAM-SP incubation, just before adding the competing ligand, to 100%. The biphasic competition patterns were analysed using the OneToTwo (1:2) model to quantify the fractions and rates of the rapid and slow releasing FAM-SP fractions.

To validate the interaction characteristics of ^125^I-labelled ligands to NK1-R expressing cell lines, the OneToOneDepletion model was applied as appropriate when working with high affinity binders and low, sub-nanomolar, ligand concentrations in the assay [52].

### 2.6 Simulations

Simulations to investigate the state of the receptor, e.g., free, bound in low affinity or high affinity/stabilized state as function of interaction time were performed using Microsoft excel following the interaction schemes as described in Eq. (Eq. 2) and Eq. 3 using the kinetic rate constants obtained from fitting experimental data to the same models.

## 3 Results

### 3.1 The interaction between NK1-R and FAM-labelled substance P

To develop a competition assay for screening potential NK1-R targeting ligands, the interaction of NK1-R with its endogenous ligand, SP was first characterized. To this end, SP labelled with fluorescein amidite (FAM) was incubated at various concentrations with NK1R-HEK293 and NK1R-U2OS cells while the fluorescence signal of bound FAM-SP was monitored over time using LigandTracer Green. Interaction curves of FAM-SP with NK1R-U2OS cells are shown in Fig. 3. The interaction is not well described by a 1:1 interaction model as depicted with the grey curve in Fig. 3. By visually comparing experimental data and the 1:1 model during the last phase of the curves, i.e., where FAM-SP is removed to monitor the dissociation process, it is seen that dissociation is biphasic meaning that a fraction of FAM-SP readily dissociates while another fraction releases more slowly. Apparently, there are two populations of FAM-SP that release with different rate constants. In view of ligand binding mechanism when interacting with GPCRs, there are two suitable models available to describe a biphasic release of FAM-SP from NK1-R; the 1:2 model that assumes that there are two interaction modes with different rates and affinities and the so-called two state model (1:1*) that assumes that FAM-SP binds in low affinity state that evolves into a stabilized second state. As can be seen in Fig. 3, both models fit equally well with the experimental data, and the fitted curves from the 1:2 and 1:1* models can hardly be distinguished.

**Fig. 3:**
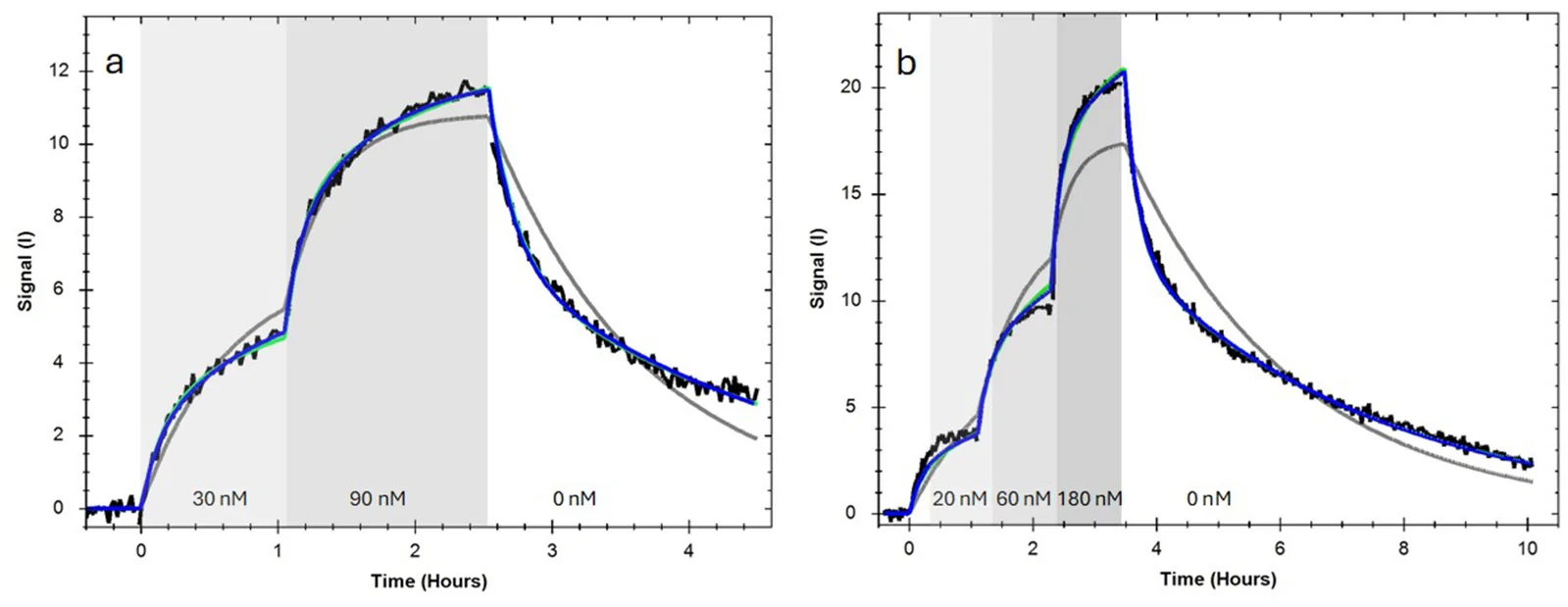
Experimental data (black) on NK1R-USO2 cells, where FAM-SP was incubated at 30 and 90 nM (a) or 20, 60 and 180 nM (b) followed by a dissociation phase. The results from fitting the data with a 1:1 model (Grey), 1:2 model (dark blue), and 1:1* model (green) are also shown.

Obtained values for the rate constants and affinities are presented in Table 1. Both the 1:2 and 1:1* models predict that there is an interaction with affinity around 100 nM (K_D1_ in Table 1). For the FAM-SP interaction with NK1R-HEK293 cells, this affinity is roughly 2-fold stronger compared to NK1R-U2OS cells and this stronger affinity results from enhanced recognition of NK1-R by FAM-SP. The 1:2 model predicts a second, stronger interaction that has an affinity around 20 nM for both NK1R-HEK293 and NK1R-U2OS. The weaker and stronger interactions predicted by this model are equally abundant. Results from applying the 1:1* model predict that binding is followed by a stabilization process to a state from which no dissociation occurs, e.g., a locked interaction on the cell surface or internalization of the FAM-SP/NK1-R complex, unless it reverses to the less stable state. According to the 1:1* model, the K_D2_ value for NK1-R/FAM-SP complex indicates that, in equilibrium, the non-stabilized fraction is half of the stabilized fraction. From the rate constants, k_a2_ and k_d2_, it can be inferred that it takes several hours before this equilibrium is reached.

**Table 1:** Results from fitting the 1:2 and 1:1* models to experimental data performed with HEK293-NK1R and USO2-NK1R cells. Average values together with the coefficient of variation (CV) in percentage are given. For both cell lines, nine RT-CBA assays were performed using various incubation times and FAM-SP concentrations. Note that for the second interaction, the units for k_a2_ and K_D2_ are model dependent. For the results from the 1:2 model, also the fraction of this second, higher affinity, interaction is given.

Model	Cell line	k_a1_ (s^−1^.mol^−1^)	k_d1_ (s^−1^)	K_D1_ (nM)	k_a2_ (s^−1^.mol^−1^)	k_d2_ (s^−1^)	K_D2_ (nM)	Fraction 2
1:2	HEK293-NK1R	5.1 x 10^4^ (66%)	1.4 x 10^−3^ (78%)	60 (115%)	0.87 x 10^4^ (84%)	8.2 x 10^−5^ (54%)	22 (107%)	57% (19%)
1:2	U2OS-NK1R	1.4 x 10^4^ (67%)	1.6 x 10^−3^ (38%)	171 (60%)	0.64 x 10^4^ (59%)	7.9 x 10^−5^ (49%)	17 (70%)	45% (25%)
					**k**_**a2**_**(s**^**−1**^**)**	**k**_**d2**_**(s**^**−1**^**)**	**K**_**D2**_	

1:1*	HEK293-NK1R	2.1 x 10^4^ (55%)	1.4 x 10^−3^ (76%)	87 (96%)	2.3 x 10^−4^ (60%)	8.2 x 10^−5^ (44%)	0.51 (54%)	
1:1*	U2OS-NK1R	1.2 x 10^4^ (45%)	1.2 x 10^−3^ (27%)	130 (48%)	1.9 x 10^−4^ (33%)	9.0 x 10^−5^ (43%)	0.49 (56%)	

Although the variation between replicates is slightly lower when applying the 1:1* model compared to the 1:2 model, there is no clear indication that one of these two models describe the interaction process better. InteractionMap analysis confirms the complexity in interaction patterns showing two interactions that are connected, indicative for a transition between these two interactions during the experiment (Supplementary Information, Fig. S2e). To further investigate the interaction mechanism, control experiments with pertussis toxin, to inhibit G-protein coupling, and fixated cells to inhibit cellular processes such as G protein coupling, internalization and receptor mobility were performed (Supplementary information, Fig. S2a–f). Results indicated that the low affinity interaction (K_D1_) is more abundant upon pertussis toxin treatment and even more so upon fixation, but the stable interaction (K_D2_) is present as well and thus not only induced by FAM-SP binding. This implies that both interactions are present when starting regular binding assays but that the strong interaction is getting more abundant during FAM-SP binding and that the actual interaction process is a combination of the 1:2 and 1:1* models. Regardless of the exact interaction mechanism, the FAM-SP interaction with NK1-R is characterized by a slow, and a rapid dissociating complex.

### 3.2 Competition assay design part I

For competition screening purposes, both slow and fast dissociating complexes are relevant for getting both fast-onset and long-lasting competitive binding. Receptor occupancy is steered by the ligand concentration in relation to the affinity reaching 50% occupancy in equilibrium when the ligand concentration is equal to the affinity value and fluctuating between 10% and 90% occupancy with 10-fold lower or higher ligand concentrations, respectively. In view of the affinity values derived for FAM-SP interactions with NK1-R to U2OS when applying the 1:2 model, a 90 nM concentration was chosen to ensure that both the low and high affinity interactions are targeted.

To better understand how the various states of NK1-R expressed on cells can evolve over time when interacting with FAM-SP, the unbound, weakly bound, and strongly bound fractions are plotted in Fig. 4 based on simulating the interactions for the 1:2 and 1:1* models using the values presented in Table 1 for interactions with U2OS cells. As can be seen from Fig. 4, regardless of the interaction process, the three NK1-R states are present in significant quantities (>20%) after incubation with 90 nM FAM-SP for 1 h. Therefore, competition experiments were performed by adding 90 nM of potential competing ligands after this 1-h incubation.

**Fig. 4:**
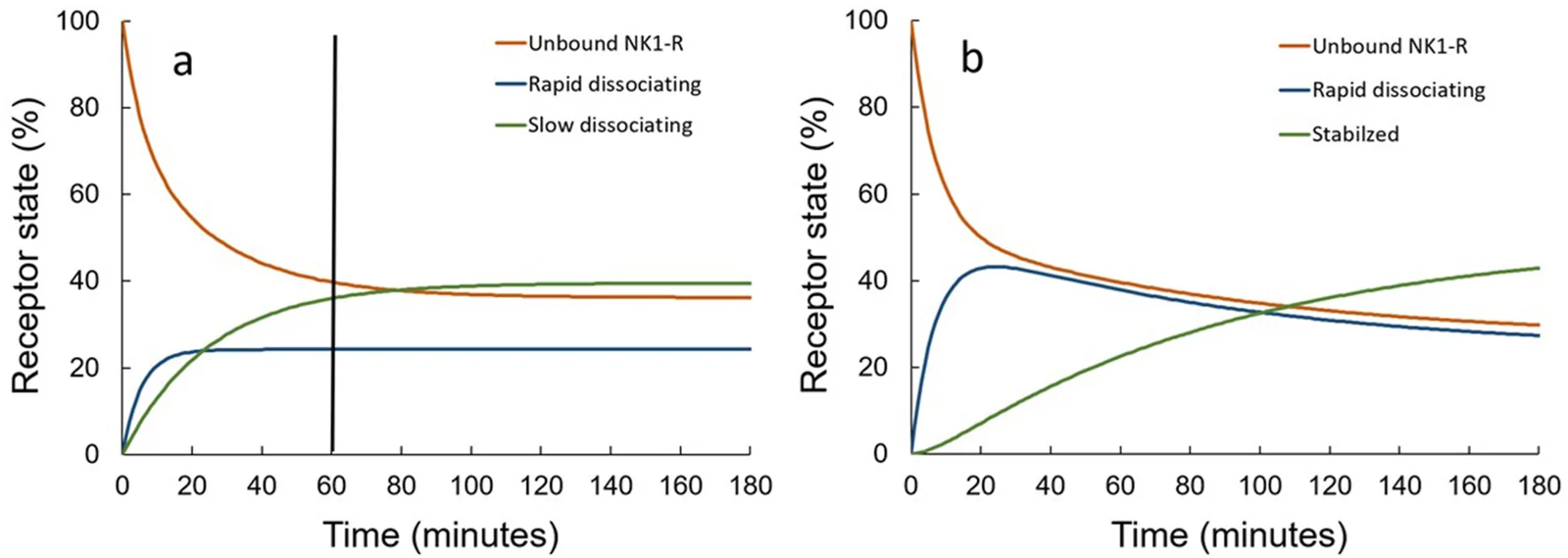
Simulation of a 1:2 interaction (a), and 1:1* interaction with stabilized FAM-SP/NK1-R complexes (b) when incubating NK1R-U2OS cells with 90 nM FAM-SP and using rate constants for association, dissociation, stabilization, and destabilization obtained from fitting these processes to experimental data. Orange: The relative quantity of unbound NK1-R, Blue: rapid dissociating FAM-SP/NK1-R complexes, Green: slowly dissociating complexes (1:2 model, Fig. 4a) or stabilized complexes (1:1* model, Fig. 4b) as function of incubation time. As indicated by the vertical lines, after 60 min, the three states are all above 20% regardless of the interaction mechanism, and this time point was chosen to start competition.

### 3.3 Competition assay design part II

For competition assay design, self-competition by the endogenous ligand SP was investigated, where NK1R-U2OS Cells were first incubated with 90 nM FAM-SP for roughly 1 h to reach a state in which the three NK1-R states; unbound, weakly bound, and stably bound to FAM-SP, were present in significant quantities. Then, 90 nM of a competing agent (in this case unlabelled SP) was added while leaving 90 nM FAM-SP in the medium, and displacement and/or replacement of FAM-SP was monitored for at least an hour (Black). An example of the resulting competition curve is shown in Fig. 5. The addition of 90 nM unlabelled SP after 1 h incubation with FAM-SP resulted in a rapid release of 70% FAM-SP (CV = 4% for triplicate). FAM-SP did not show binding to the background area coated with proteins from FBS or to U2OS wild type cells (Supplementary information, Fig. S3a and b). To illustrate the effect choosing the FAM-SP concentration in relation to the affinities, the result of a competition assay with 30 nM FAM-SP is shown in Fig. S3a where binding signal is lower and, upon adding 30 nM unlabelled SP, only competition with the high-affinity state is observed. The competition assay could not be analysed with a standard competition model in TraceDrawer evaluation software which assumes a 1:1 binding interaction for both the labelled and competing ligands. The resulting interaction pattern of the competition assay was, however, well described by a 1:2 model as can be seen in Fig. 5a. Applying this model to the curves quickly reveals the rapid initial displacement rate (k_rd_), slow replacement rate (k_sr_), and the fraction FAM-SP that is slowly replaced (F_sr_) as visualized in Fig. 5a by plotting both interactions obtained from the 1:2 fit.

**Fig. 5:**
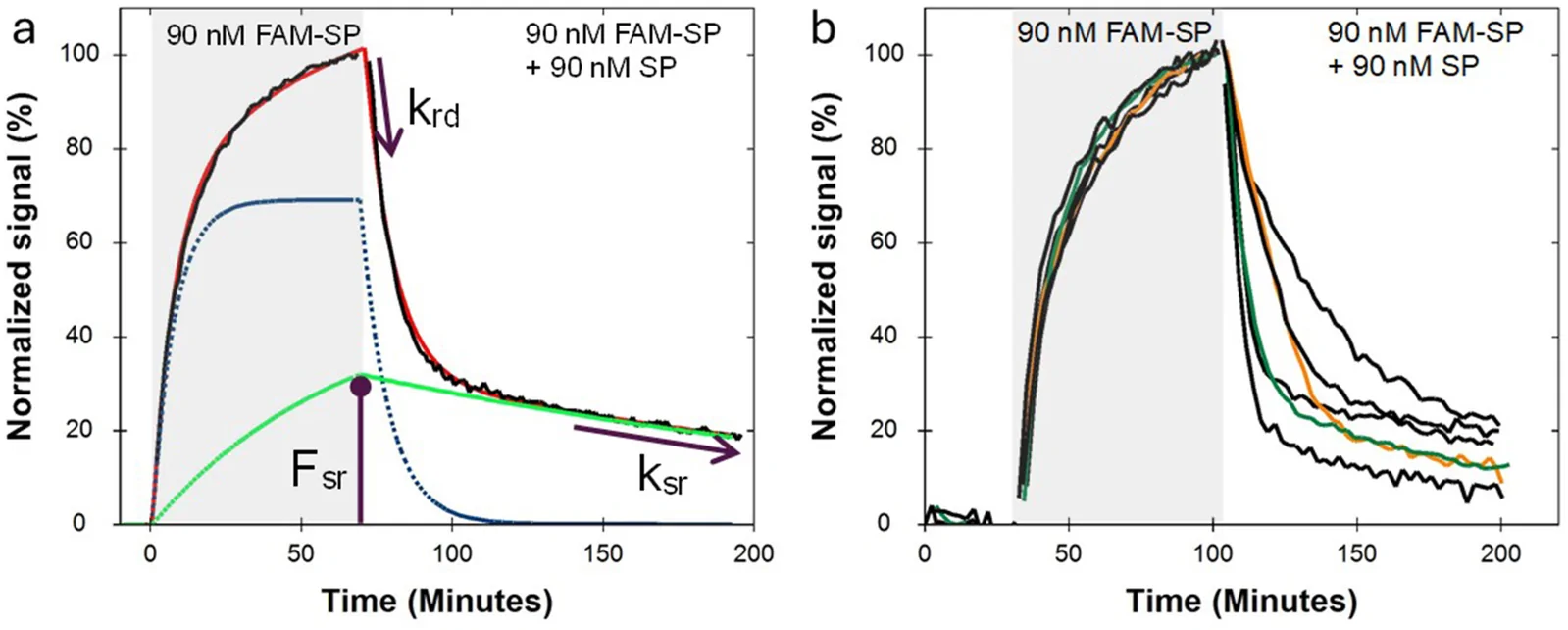
a) FAM-SP self-competition by unlabelled SP. The resulting curve is well described by a 1:2 model (red) that divides the competition process into a FAM-SP fraction with a rapid displacement rate (Blue) referred to as k_rd_ and another fraction with a slow replacement rate (Green) referred to as k_sr_. The signal, prior to adding the competitor, is normalized to 100% to easily display the percentage of the stable fraction at the start of competition which is referred to as F_sr_. b) Representative FAM-SP competition assay results. The black curves are examples of displacement curves of a few ligands (At 200 min from top to bottom: PN951, PN017, PN622, PN362) compared to that of SP (Green) and aprepitant (Orange).

The average rapid displacement rate for three replicates amounted to 2.3 × 10^−3^ s^−1^ (CV = 12%). This rapid release rate was almost 2-fold faster than the rapid FAM-SP dissociation rate (see Fig. 3 and Table 1) meaning that the addition of 90 nM SP not only inhibits rebinding of FAM-SP that is still present but also actively displaces bound FAM-SP. The slow releasing FAM-SP fraction has a more than 20-fold slower average releasing rate of 1.0 × 10^−4^ s^−1^ (CV = 42%), a value that is very close to the dissociation or destabilization rate of the stably bound fraction (k_d2_) as reported in Table 1. This indicates that SP replaces stably bound FAM-SP when it is released and inhibits FAM-SP rebinding.

### 3.4 NK1-R binding ligand screening

The designed competition assay was used to screen 60 synthetic ligands as well as the clinically used NK1-R inhibitor aprepitant and SP as references. Many ligands showed displacement patterns similar to that of SP but with some variation in the initial rapid displacement rate (k_rd_), the fraction that is slowly replaced (F_sr_), and the rate in which this stable fraction is replaced (k_sr_). As an example, competition assay results for a few of these ligands, together with that of the natural ligand (SP) and aprepitant, are shown in Fig. 5b. Curves for the competition assay for all 60 ligands are shown in supplementary information (Fig. S4).

SP and aprepitant displaced FAM-SP rapidly. It is assumed that the competing ligands should also displace FAM-SP rapidly to compete with SP binding *in vivo*. The FAM-SP displacement rates in the first 10 min, relative to that of SP, for all ligands are presented in Fig. 6. Most ligands displaced FAM-SP with rates in the same range as that of SP, i.e., from 10^−3^ to 10^−4^ s^−1^ meaning that the displacement half-life is ranging from 10 min to 2 h. Aprepitant displaced FAM-SP a little more than 3-fold slower than SP. To focus on ligands that have the potential to displace FAM-SP faster or similar to that of aprepitant, an initial ligands selection was made by excluding all ligands that have more than a 4-fold lower displacement rate than SP. This cut off level is depicted by the red line in Fig. 6. This initial selection resulted in the exclusion of 33 ligands from further evaluation. Of these 33 ligands, only six ligands hardly displaced FAM-SP with rates that were at least 1000-fold lower than SP.

**Fig. 6:**
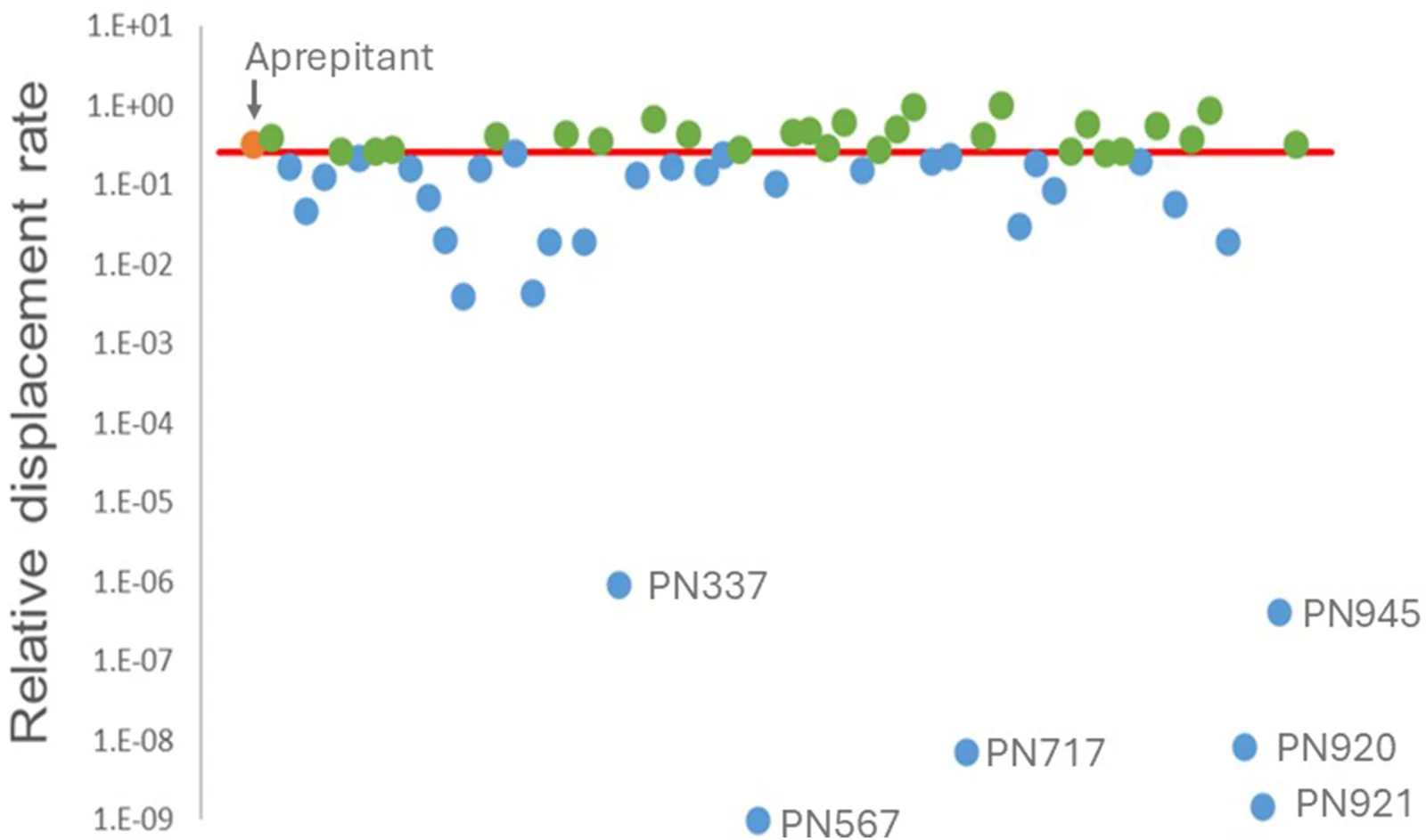
Scatter plot showing displacement rates during the first 10 min relative to that of SP for 60 potential competing ligands and aprepitant (orange), plotted on a logarithmic scale to accommodate the wide range of observed values. Ligands with a more than four-fold lower displacement rate than SP (depicted by the red horizontal line) are coloured in blue and excluded from further evaluation. The 27 new ligands that were above this level (green) were selected for further evaluation.

The 1:2 model was applied to the remaining 27 potential competing ligands and results of this analysis are presented in Fig. 7, where the rapid displacement rate (k_rd_) relative to that of aprepitant is given in percentage. The ability of the ligands to replace stably bound FAM-SP relative to aprepitant is represented by the bar colour with blue denoting that the replacement rate of the stable fraction (k_sr_) is faster than that of aprepitant and green denoting that the remaining stably bound fraction (F_sr_) is small, i.e., between 15 and 30% while the smallest fraction (15%) was observed for aprepitant itself. Actual values for the rapid displacement rate (k_rd_) and replacement rate (k_sr_) relative to aprepitant and the remaining stably bound fraction (F_sr_) are given in supplementary information (Table S5).

**Fig. 7:**
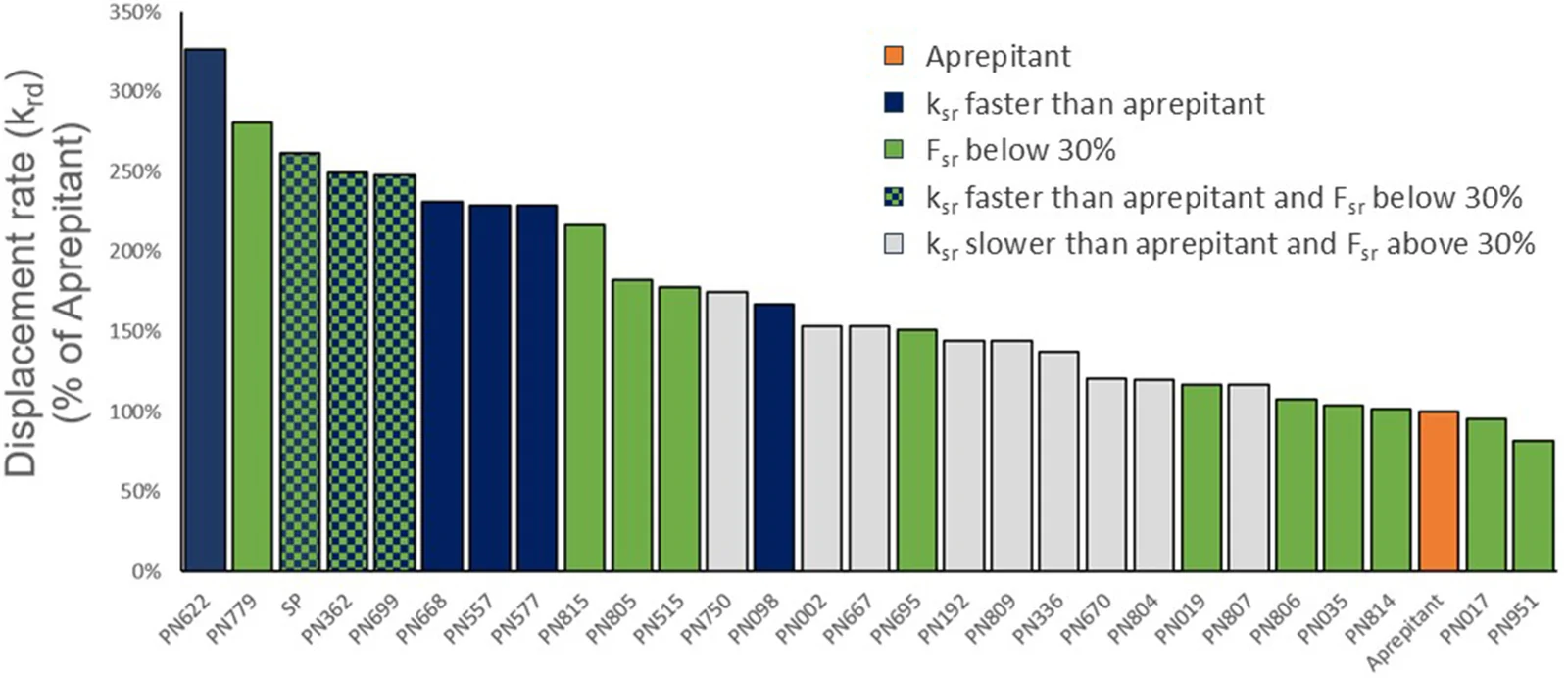
Results from analysing competition assay relative to aprepitant. The y-axis represents the relative rapid initial displacement rate (k_rd_) with the k_rd_ of aprepitant set to 100%. Bars are coloured according to their ability to replace the stable FAM-SP fraction. Blue: k_sr_ is faster than aprepitant, Green: F_sr_ at the start of displacement is between 15 and 30%, i.e., less than two-fold higher than that of aprepitant. Only two compounds, PN363, and PN699 as well as SP have a checkered bar indicating that the remaining stable fraction is below 30% and is replaced rapidly. Grey bars represent the opposite, i.e., k_sr_ is slower than Aprepitant and F_sr_ is larger than 30%.

In Fig. 7, the 27 selected ligands together with SP and aprepitant divide into three groups. On the right end of x-axis, a few ligands, i.e., PN017, PN019, PN035, PN814, and PN951 behave similar to aprepitant. Actual displacement patterns for PN017, PN951 and aprepitant were shown in Fig. 5b. These ligands have comparable initial rapid displacement rates after which a relatively small fraction of stably bound FAM-SP remains, as indicated by the green bar colour. Nevertheless, none of these ligands replaced the stable fraction faster than aprepitant. In the middle, a group of ligands grouped together that have a more rapid initial displacement rate than aprepitant but were less effective in replacing stably bound FAM-SP with both a larger fraction and slower replacement rate of this stable fraction as denoted by grey bars. On the left end of x-axis, ligands are found that not only showed much faster displacement than aprepitant but also showed either a faster replacement of stably bound FAM-SP (blue bars), a relatively small stable fraction (green bars), or both (checkered bars). Actual competition patterns of a few of these ligands, i.e., PN622, PN362 and SP were presented in Fig. 5b. Again, none of the ligands were equally good in reducing the stable bound fraction (F_sr_) as aprepitant. Only two competing ligand, i.e., PN362 and PN699 displaced both the weakly and strongly bound fraction of FAM-SP faster than aprepitant while F_sr_ is below 30%. For PN362, F_sr_ was slightly higher (21%) than that of aprepitant (15%) and PN363 the only newly synthesized competing ligand that was able to replace all FAM-SP within 3 h, as does self-competition with SP.

### 3.5 Assay validation

To validate the ligand screening assay, the interaction between ligands and various cancer cell lines endogenously expressing NK1-R, overexpressing NK1-R, and NK1-R negative cells were investigated. A standard real-time cell binding assay (RT-CBA) was performed to monitor the binding interactions. For this validation, the iodine atom in the ligands was replaced with the radioactive isotope Iodine-125 for monitoring the interaction using LigandTracer Grey. Iodine-125 is commonly used as a proxy for iodine-124 or iodine-131, is readily available, and has a long half-life to support extended experiments without the need for frequent radiolabelling. Two ligands were selected for assay validation: ^125^I-PN622, a ligand that caused a faster FAM-SP release of both the weak and stably bound fraction than substance P and ^125^I-PN951, a ligand with a similar competition pattern as aprepitant. Interaction curves with endogenous NK1-R expressing cell lines are shown in Fig. 8A and B while interaction curves with overexpressing cell lines are presented in Fig. 8C and D. ^125^I-PN622 did not show significant binding to U2OS cells transfected with another GPCR; the somatostatin receptor 2A (SSTR), as presented in Fig. 8c. Neither PN622 nor PN951 showed binding to the background position of the dish that is coated with proteins from FBS in the medium (data not shown). Resulting values for the affinity and interaction rate constants are presented in Table 2.

**Fig. 8:**
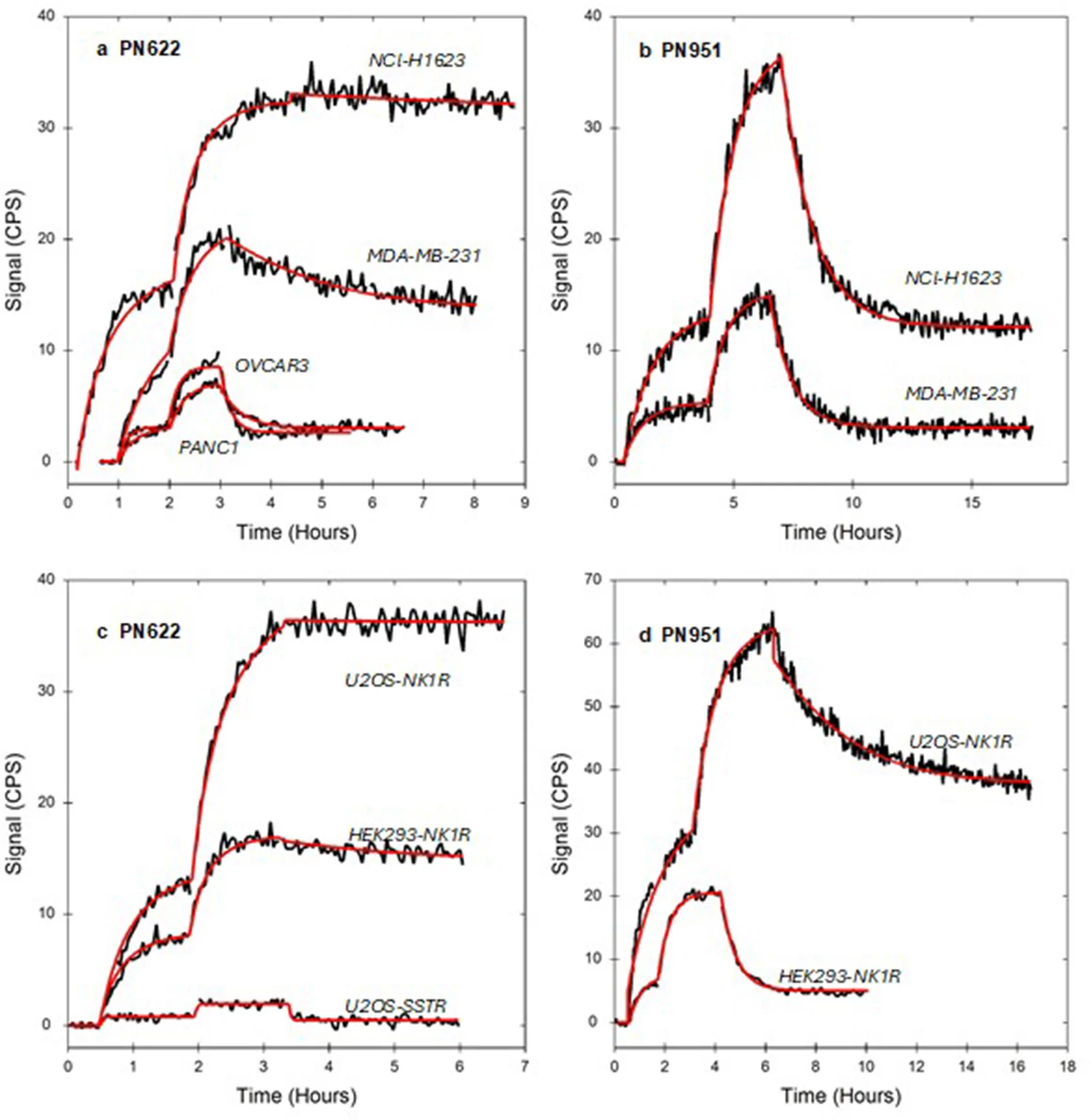
Real-time cell binding assays for various ligands interacting with endogenous NK1-R cell lines (a & b) and transfected NK1-R cell lines (c & d) and U2OS transfected with SSTR (c). Experimental curves are in black and result from fitting data with a 1:1 depletion model in red. a) PN622 (40 and 100 pM) with MDA-MB-23, OVCAR3, and PANC1 cells, b) PN951 (1 and 3 nM) with NCI-H1623 and MDA-MB-231 cells. c) PN622 (40 and 100 pM) with U2OS-NK1R, U2OS-SSTR, and HEK293-NK1R cells and d) PN915 (1 and 3 nM) with U2OS-NK1R and HEK293-NK1R cells.

**Table 2:** The affinity (K_D_), association rate constant (k_a_) and dissociation rate constant (k_d_) for interaction of^125^I-labelled ligands^125^I-PN622 and^125^I-PN951 with various cell lines. ND = not determined.

Cell line	^125^I-PN622			^125^I-PN951		
	K_D1_ (pM)	k_a1_ (s^−1^.mol^−1^)	k_d1_ (s^−1^)	K_D1_ (pM)	k_a1_ (s^−1^.mol^−1^)	k_d1_ (s^−1^)
**U2OS-NK1R**	0.05	5.6 x 10^6^	2.6 x 10^−7^	300	9.3 x 10^4^	2.8 x 10^−5^
**HEK293-NK1R**	1.4	11.8 x 10^6^	1.6 x 10^−5^	5590	6.2 x 10^4^	3.5 x 10^−4^
**NCl-H1623**	0.26	7.3 x 10^6^	1.9 x 10^−6^	4910	2.8 x 10^4^	1.6 x 10^−4^
**MDA-MB-231**	7.2	5.5 x 10^6^	4.0 x 10^−5^	9720	2.5 x 10^4^	2.4 x 10^−4^
**OVCAR3**	185	2.9 x 10^6^	5.3 x 10^−4^	ND	ND	ND
**PANC1**	985	9.9 x 10^5^	9.8 x 10^−4^	ND	ND	ND

Comparing the interaction patterns of ^125^I-PN622 with ^125^I-PN951 shows that ^125^I-PN622 clearly releases slower and is thus more stably captured by the cells. Interestingly, the release is slower when binding signals are higher, indicating that binding is more stable when receptor expression levels are higher. This results in the strongest binding for ^125^I-PN622 interacting with the high expressing U2OS-NK1R cells resulting in an affinity of 50 fM. ^125^I-PN951 also shows the highest affinity for U2OS-NK1R cells but with a 6000-fold weaker affinity than ^125^I-PN622. For the interaction of both ligands with MDA-MB-231, the binding strength of ^125^I-PN622 is also much higher than that of ^125^I-PN951.

The difference in affinity between the compounds is both the result of better recognition, as reflected by the 60- to 260-fold faster association rates for ^125^I-PN622 than ^125^I-PN951 when comparing interactions on the same cell line, and 6- to 110-fold slower dissociation rates as reflected by the dissociation rate constants. The cell-line dependent difference for both ligands is primarily caused by a slower dissociation of the ligands when binding signals are higher suggesting that high receptor expression levels increase ligand retention.

## 4 Discussion

Ligand binding competition occurs when two or more ligands compete for the same binding site on a protein, with the outcome influenced by their relative affinities and concentrations. This principle is commonly used in competition binding assays, where displacement of a labelled probe by an unlabelled ligand allows measurement of binding affinity and selectivity. In complex interaction systems like GPCRs, such as the NK1-R with SP as its agonist, competition can be more intricate. The complexity of this binding mechanism corresponds to the conformational dynamics and ligand interactions mechanism that has been described for NK1-R [7,8] and GPCRs in general [[31], [32], [33], [34], [35], [36]].

Owing to the rapid enzymatic degradation, non-selective receptor binding, and broad biological activity of Substance P, structurally modified analogues, NK1-R antagonists, or their radiolabelled derivatives are generally favoured for theranostic applications, as they offer enhanced metabolic stability, receptor specificity, and therapeutic efficacy. In this study, LigandTracer was used to screen the best candidates among 60 synthetically produced NK1-R binding ligands in real-time on live cells. First, the interaction of FAM-labelled SP was evaluated on both live and fixated cells, which confirmed the expected complex interaction pattern with NK1-R overexpressing U2OS and HEK293 cells. The complex interaction indicated that there are two states; one from which FAM-SP readily dissociates and another state that retains FAM-SP with high affinity while the high affinity state becomes more abundant upon incubation with FAM-SP. Also, FAM-SP interaction patterns to fixated and pertussis toxin treated cells and InteractionMap analysis (Fig. S2 d and f) support that both the low and high affinity interactions are present before starting incubation of NK1R-U2OS cells with FAM-SP and the abundancy of the high affinity component increases upon incubation time. These observations are similar to those reported for interaction patterns of ^177^Lu-DOTATE interacting with the somatostatin receptor [35]. By systematic inhibiting G-protein coupling, internalization, recycling, and synthesis it was seen that the G-protein coupled and uncoupled receptors are both present on the cell surface. Upon adding ^177^Lu-DOTATE, the G-protein coupled high-affinity state becomes more abundant initially. A substantial fraction of the ligand is located intracellularly with a rapid exchange between intra- and extracellular GPCRs resulting in binding pattern that are only marginally affected by the internalization and recycling process. Eventually, the weaker uncoupled GPCR state becomes more abundant again after a few hours of ligand exposure.

Based on the complex interaction pattern, a competition assay was developed where FAM-SP binding leads to significant NK1-R occupancy of the various states. Displacement and/or replacement of FAM-SP was then monitored as function of time upon addition of unlabelled ligands at an equimolar concentration. Self-competition with unlabelled SP revealed that 70% FAM-SP rapidly detached from NK1R-U2OS cells with a rate that was 2-fold higher than the FAM-SP dissociation rate. This means that SP actively displaces 70% of FAM-SP. The remaining 30% FAM-SP was released with a 20-fold slower rate which was similar to the dissociation rate of the stably bound FAM-SP fraction, indicating replacement rather than displacement. During this competition phase, FAM-SP and SP were both present in equimolar concentrations. Nevertheless, FAM-SP was released from the cells at a higher or similar rate as the dissociation rate in the absence of competing ligand suggesting that SP effectively blocks FAM-SP rebinding. Apparently, the FAM label strongly reduces the affinity of SP for NK1-R which is supported by the more than 100-fold stronger affinities that have been reported for SP binding to the two receptor states, i.e., 50 and 500 pM [8] compared to the values around 20 and 100 nM obtained for FAM-SP as presented in Table 1.

In the first selection process, 27 out of 60 ligands were selected that displaced FAM-SP with a faster or similar rate than aprepitant during the first 10 min. To analyse the competition assay results from these 27 ligands, a 1:2 model was applied to extract multiple parameters, i.e., the initial rapid displacement rate (k_rd_), the fraction that is slowly replaced (F_sr_), and the rate in which this stable fraction is replaced (k_sr_). Ranking the ligands on these three parameters revealed that the ligands were divided into three groups with one group clustering around the natural ligand SP and another group clustering around the antagonist aprepitant.

When examining the chemical structures of the ligands, there is no clear structure-activity relationship between the three groups. Both ligand PN622 and PN362, that cluster around SP, have an H-atom in the R1 position but this also applies for PN035 that clusters around aprepitant. For several other ligands that cluster around aprepitant (PN019, PN814, PN017, and PN951), the R1 group is a CH_2_-triazolone as is the case for aprepitant itself.

To validate the assay, the affinity of two ligands, interacting with endogenous NK1-R expressing epithelial cell lines and NK1-R overexpressing U2OS and HEK293 cell lines was evaluated. The radiolabelled ligands ^125^I-PN622 that scored highest on the rapid displacement rate (k_rd_) and high on the slower replacement rate (k_sr_) showed an affinity which is substantially stronger than affinities reported for a previously published NK1-R antagonist labelled with ^67^Ga (12 nM) or ^64^Cu (15 nM) [19] or ^177^Lu-labelled ligands that ranged from 2 to 10 nM [21]. Interestingly, the affinity varied strongly for different cell lines ranging from 985 pM for PANC1 cells to 50 fM for U2OS cells. Affinities were stronger for interaction curves with higher signals indicating that the affinity correlates with NK1-R expression levels. Affinity values for interactions with endogenous NK1-R expressing cells were in line with an IC_50_ value of 100 pM reported for aprepitant [53] and 60 pM reported for a lead tricyclic compound [22].

In contrast to FAM-SP interaction with NK1-R, the interaction of PN622 with NK1-R was characterized by a simple 1:1 interaction, characteristic for antagonists rather than the complex interaction mechanism typically observed for agonist GPCR binders. The competition pattern of several ligands that group together with the natural ligand SP, raises the question whether some of these ligands also bind in an agonistic manner, potentially promoting tumorigenesis. Ongoing studies suggest that PN622 may act as an antagonist, but further characterization of PN622 and other top candidates is necessary to evaluate their binding mode and cell line–dependent affinities based on receptor expression levels. These aspects will be addressed in future studies.

## 5 Conclusion

A new method was developed to quantify both the rapid displacement (actively forcing labelled SP to release) and the slow replacement of stably bound labelled SP (binding to NK1-R when labelled SP releases) resulting in lead candidates that have high affinity for NK1-R expressing cells. One of the lead candidates, PN622, displayed very high affinity to NK1-R expressing cells and is a promising candidate for theranostic applications for treating and imaging neural tumours.

## CRediT authorship contribution statement

**Hadis Westin:** Writing – review & editing, Writing – original draft, Supervision, Methodology, Formal analysis, Data curation, Visualization. **Hanna Yushchyshyna:** Data curation, Investigation. **Olga Vorontsova:** Writing – review & editing, Investigation, Data curation. **Saloni Chopra:** Writing – review & editing, Validation. **Tianqi Xu:** Writing – review & editing, Validation. **Christoffer Bengtsson:** Methodology. **Fabio Begnini:** Methodology. **Preeti Jha:** Methodology. **Ulrika Rosenström:** Writing – review & editing, Supervision. **Ulrika Yngve:** Writing – review & editing, Methodology, Supervision. **Marika Nestor:** Writing – review & editing, Supervision, Funding acquisition, Conceptualization. **Jos Buijs:** Conceptualization, Formal analysis, Methodology, Supervision, Visualization, Writing – original draft, Writing – review & editing.

## Funding

This work was supported by the Swedish Childhood Cancer Fund (Barncancerfonden) under grant number PR2023-0033; the 10.13039/501100002794Swedish Cancer Society (Cancerfonden) under grant numbers CAN 24 3485 Pj, 22 2391 S; the 10.13039/501100004359Swedish Research Council (Vetenskapsrådet) under grant number 2024-03447.

## Declaration of competing interest

The authors declare the following financial interests/personal relationships which may be considered as potential competing interests:Marika Nestor reports financial support was provided by Swedish Childhood Cancer Fund. Marika Nestor reports financial support was provided by Swedish Cancer Society. Marika Nestor reports financial support was provided by Swedish Research Council. Jos Buijs reports a relationship with Ridgeview Instruments AB that includes: board membership, employment, and equity or stocks. Hadis Westin reports a relationship with Ridgeview Instruments AB that includes: employment. Marika Nestor, Ulrika Rosenström, Preeti Jha, Christoffer Bengtsson, Ulrika Yngve, and Fabio Begnini have a patent application #WO2025008409. If there are other authors, they declare that they have no known competing financial interests or personal relationships that could have appeared to influence the work reported in this paper.

## Data Availability

Data will be made available on request.
