# Supplementary material for: Development of a GPCR-binding ligand screening method using real-time interaction analysis on live cells
Source: Biochem Biophys Rep. 2026 Sep 30;48:102818. doi: 10.1016/j.bbrep.2026.102818 (PMC13653987; doi:10.1016/j.bbrep.2026.102818)
Supplement: Supplementary file 1 [file mmc1.docx]

# Supplementary information

## S1 Ligands

PN951

PN622

Aprepitant

Fig. S1. Chemical structures of aprepitant and ligands PN622 and PN951 used to validate the developed screening assay**.**

## S2. Binding kinetics evaluation of FAM-SP on live, pertussis toxin treated, and fixated NK1-R over-expressing U2OS cells

To assess the influence of receptor mobility and interactions with cellular proteins on ligand-NK1-R interactions, control experiments were performed after pre-incubation for one hour with 400 ng/mL pertussis toxin (P2980, Sigma-Aldrich) to inhibit G-protein coupling and with fixated cells. For fixation, cells were washed after overnight incubation with PBS followed by 10 min incubation at room temperature with 10 mL methanol-stabilized, buffered (pH 7.0 ± 0.1) aqueous 4% PFA solution (VWR BDH Chemicals cat# 9713.1). Thereafter, cells were washed three times with 10 mL PBS and replaced with 3 mL CO_2_-independent medium for a real-time cell binding assay (RT-CBA).

30 nM	90 nM	0 nM

30 nM	90 nM	0 nM

b

a

**e**

**f**

**c**

**dd**

**1**

**1**

**2**

**2**

**2**

**2**

**1**

**1**

Fig. S2. a) RT-CBA of FAM-SP on live (black) and pertussis toxin treated (green) NK1R-U2OS cells and b) RT-CBA of FAM-SP on live (black) and fixed (green) NK1R-U2OS cells. For all RT-CBA experiments, the cells were incubated with 30 and 90 nM FAM-SP, followed by monitoring dissociation. The association phase was not significantly affected by either pertussis toxin treatment or fixation, but dissociation became faster, especially upon fixation. However, the dissociation remains biphasic meaning that one FAM-SP fraction releases with a faster rate than another FAM-SP fraction as observed for the experiments performed with live cells. This fast-releasing fraction is larger for pertussis toxin treated cells and even more so for fixed cells compared to live cells, indicating that the live cell environment results in a slower release, and thus a higher affinity, for FAM-SP. InteractionMap analysis of the interaction with live (c and e), pertussis toxin treated (d) and fixated (f) NK1R-U2OS cells. InteractionMap is a tool that finds the optimal combination of 1:1 interactions, with various combinations of on-rates (y-axis) and off-rates (x-axis). The FAM-SP interaction with live NK1R-U2OS cells is characterized by two peaks where the peak denoted with number 2 has a slightly slower on and a significant slower off-rate than peak 1 and thus represents the stable and slow releasing FAM-SP fraction. For the interaction with pertussis toxin treated and fixed cells, where cellular processes and mobility are inhibited, peak 1 becomes dominant but is elongated towards the more stable state indicating that this state is partly present when pertussis treating or fixating cells and thus before incubation with FAM-SP. These results indicate that receptor states giving rise to both rapidly and slowly releasing FAM-SP fractions are present prior to FAM-SP incubation, while the slow-releasing interaction increases and stabilizes over time. The peaks are extended towards each other, indicating a transition between the two states during the assay.

## S3. Competition assay design

a

b

30	90	270	810 nM FAM-SP

0 nM

20 nM

60 nM

180 nM

90 nM FAM-SP

30 nM FAM-SP

+ 30 nM SP

30 nM FAM-SP

90 nM FAM-SP

+ 90 nM SP

Fig. S3a. Standard competition assay where NK1R-U2OS cells are incubated with 90 nM FAM-SP for 1 hour followed by addition of 90 nM unlabelled SP where the signal from the target position (grey), the background position (blue), and background corrected signal (black) are shown to demonstrate that no FAM-SP binding to the background, coated with proteins absorbed from FBS in the medium, is visible. In green, the background corrected signal for a similar assay with 30 nM FAM-SP followed by addition of 30 nM SP. With this lower, 30 nM FAM-SP concentration, FAM-SP primarily binds to the high affinity NK1-R state, and the competition phase resembles that of the slow releasing fraction only.

Fig S3b. FAM-SP was incubated at 30, 90, 270, and 810 nM with wild type U2OS cells (blue) to demonstrate that FAM-SP specifically interacts with NK1-R-U2OS cells as seen in the black curve where FAM-SP was incubated with 20, 60 and 180 nM concentrations followed by a zero-concentration dissociation phase.

## S4. All competition assays

Fig. S4. Competition assay for all ligands. In all assays, NK1R-U2OS cells are incubated with 90 nM FAM-SP for 1 hour followed by addition of 90 nM of a potential competing ligand (PNxxx, or aprepitant). For comparison purposes, the dissociation of FAM-SP without any competing ligand is shown in all graphs (dark blue), alongside self-competition using 90 nM unlabelled SP (dark green).

´

## S5. Evaluation of competition assay for 27 selected ligands

The 1:2 model was applied to the 27 remaining potential competing ligands, SP and aprepitant and results are given in the Table below. Ligands are ordered on their rapid displacement rates. The ligand name is given in the first column and for each of the ligand, the second column gives the rapid displacement rate (k_rd_), relative to that of aprepitant. The ability of the ligands to replace stably bound FAM-SP is given in columns 3 and 4. Column 3 gives the replacement rate of the stable fraction (k_sr_) relative to that of aprepitant and column 4 gives the percentage of the stably bound fraction (F_sr_).

| **Ligand** | **k_rd_/k_rd-_aprepitant (%)** | **k_sr_/k_sr-_aprepitant (%)** | **F_sr_ (%)** |
| --- | --- | --- | --- |
| PN622 | 327 | 153 | 51 |
| PN779 | 281 | 58 | 16 |
| SP | 261 | 267 | 27 |
| PN362 | 249 | 175 | 21 |
| PN699 | 248 | 183 | 29 |
| PN668 | 231 | 113 | 35 |
| PN557 | 229 | 110 | 49 |
| PN577 | 229 | 110 | 35 |
| PN815 | 217 | 0 | 19 |
| PN805 | 183 | 0 | 23 |
| PN515 | 178 | 20 | 29 |
| PN750 | 175 | 5 | 34 |
| PN098 | 167 | 108 | 31 |
| PN002 | 153 | 0 | 32 |
| PN667 | 153 | 36 | 40 |
| PN695 | 151 | 0 | 28 |
| PN192 | 145 | 53 | 30 |
| PN809 | 145 | 0 | 47 |
| PN336 | 137 | 0 | 36 |
| PN670 | 121 | 30 | 41 |
| PN804 | 120 | 31 | 44 |
| PN019 | 117 | 0 | 25 |
| PN807 | 117 | 0 | 44 |
| PN806 | 108 | 20 | 21 |
| PN035 | 104 | 6 | 26 |
| PN814 | 102 | 69 | 24 |
| Aprepitant | 100 | 100 | 15 |
| PN017 | 96 | 0 | 22 |
| PN951 | 82 | 26 | 21 |
